# Screening and Verification of Molecular Markers and Genes Related to Salt-Alkali Tolerance in *Portunus trituberculatus*

**DOI:** 10.3389/fgene.2022.755004

**Published:** 2022-02-08

**Authors:** Wen Zhang, Xiao Yan Zhao, Jie Wu, Ling Jin, Jianjian Lv, Baoquan Gao, Ping Liu

**Affiliations:** ^1^ Key Laboratory of Sustainable Development of Marine Fisheries, Ministry of Agriculture, P.R.China, Yellow Sea Fisheries Research Institute, Chinese Academy of Fishery Sciences, Qingdao, China; ^2^ Function Laboratory for Marine Fisheries Science and Food Production Processes, Qingdao National Laboratory for Marine Science and Technology, Qingdao, China; ^3^ College of marine technology and environment, Dalian Ocean University, Dalian, China

**Keywords:** BSA, INDEL, Portunus trituberculatus, salt-alkali, SNP

## Abstract

Salt-alkali tolerance is one of the important breeding traits of *Portunus trituberculatus*. Identification of molecular markers linked to salt-alkali tolerance is prerequisite to develop such molecular marker-assisted breeding. In this study, Bulked Segregant Analysis (BSA) was used to screen molecular markers associated with salt-alkali tolerance trait in *P. trituberculatus*. Two DNA mixing pools with significant difference in salt-alkali tolerance were prepared and 94.83G of high-quality sequencing data was obtained. 855 SNPs and 1051 Indels were firstly selected as candidate markers by BSA analysis, out of which, 20 markers were further selected via △index value (close to 0 or 1) and eight of those were successfully verified. In addition, based on the located information of the markers in genome, eight candidate genes related to salt-alkali tolerance were anchored including ubiquitin-conjugating enzyme, aspartate–tRNA ligase, vesicle-trafficking protein, and so on. qPCR results showed that the expression patterns of all these genes changed significantly after salt-alkali stress, suggesting that they play certain roles in salt-alkali adaptation. Our results will provide applicable markers for molecular marker-assisted breeding and help to clarify the mechanisms of salt-alkali adaptation of *P. trituberculatus*.

## Introduction

*Portunus trituberculatus* (Juan and Gaoli, 2021; Ye and Fangmin, 2021), which belongs to Crustacea, Decapoda, Portunus family, commonly known as Portunus, is an important breeding species along the coast of China (Wu, 2014). Saline-alkali waters are a form of worldwide low-yield water resources, and there are about 690 million mu of low-lying saline-alkali waters in inland China alone. However, due to its high salinity, high alkalinity, high pH, and complex ion composition (Li, 2020), common aquatic animals cannot survive and reproduce in this environment suitably. The use of this type of water resource is thus, greatly hindered. High salt-alkali stimulation will reduce the feeding, metamorphosis, and survival rates of juvenile crabs (Hu et al., 2014). Therefore, the salt-alkali tolerance trait is one of the important breeding traits of *P. trituberculatus*.

At present, research on salt-alkali tolerance in aquatic animals mainly focus on fishes, especially on species with natural resistance to salt-alkali, such as *Oncorhynchus clarkihenshawi* (Wilkie and Wright, 1994), and *Chalcalburnus tarichi* (Arabacı and Sarı, 2004). Studies of molecular physiological mechanisms have been carried out on *Oreochromis niloticus* (Biao and Li, 2012; Zhao and Wu, 2016) and *Oryzias latipes* (Yao and Wang, 2012). Some important ion transport genes including Na^+^ -K+-ATPase, *slc4a1*, *slc4a4*, and *slc26a6*, were cloned and proved to play an important role in salt-alkali tolerance (Whittamore, 2012; Wood and Bergman, 2012). Compared with fish, less studies documented salt-alkali stress in aquatic crustaceans (Fu, 2002; Fu-yi et al., 2005; Li et al., 2021). Out of those, most studies are similar to the work in fish, focusing on expression of ion transport-related genes (Na^+^/K^+^-ATPase, HSP, proPO) (Shen et al., 2014; Xu et al., 2020) and the analysis of enzyme activity (ACP, AKP, SOD) (Liu, 2016; Xu et al., 2020) after salt-alkali stress. However, just a few cases were reported on whether crustaceans have special salt-alkali tolerance genes.

Molecular markers are prerequisite for marker-assisted breeding. Due to the important economic value of *P. trituberculatus*, some growth-, sex-, and immunity-related markers have been discovered and verified based on quantitative trait locus (QTL) and association analysis (Lv et al., 2017; Lv et al., 2018; Zhou and Zhao, 2019). Recently, in terms of environmental adaptation, some salinity-related molecular markers have also been reported (Lv et al., 2019). However, there were no reports on the development of salt-alkali tolerance molecular markers in crabs.

In this study, we explored the salt-alkali tolerance molecular markers in *P. trituberculatus* by the Bulked Segregant Analysis (BSA) strategy. In addition, based on the location information of the salt-alkali tolerance molecular markers on genome, we tried to anchor genes related to salt-alkali adaptation in crab. Our results will provide applicable markers for molecular marker-assisted breeding and help to clarify the mechanism of salt-alkali adaptation of *P. trituberculatus*.

## Materials and Methods

### Experimental Material

The crab used in this study was cultured in Changyi Haifeng Aquaculture Co., Ltd., Weifang City, Shandong Province, China. Six hundred healthy crabs were selected and randomly divided into three groups (experimental, control, and sample), and the average weight was 55.28 g. During the temporary rearing (holding for 7 days), the water temperature was maintained at 22 ± 1 °C and pH 8.2 ± 0.5. Seawater of the experimental group and sample group was adjusted to 16.7 mmol/L by adding carbonate, and for the control group, normal seawater was used. Then, salt-alkali stress was observed every 2 h and dead individuals were collected in time. The experiment lasted for 72 h, until the survival rate of the experimental group was 5%, while no death was recorded in the control group. The first 20 individuals that died were from the saline-alkali-sensitive group (S) and the last 20 individuals that survived were from the saline-alkali-tolerant group (T). Muscle tissues from the crabs were collected and stored in liquid nitrogen. In the sampling group, gill tissues were sampled at 0, 6, 12, 24, 48, and 72 h after stress, and stored in liquid nitrogen for cDNA for qPCR detection analysis.

### Library Construction and Sequencing

DNA degradation and contamination was monitored on 1% agarose gels. DNA purity was checked using NanoPhotometer^®^ spectrophotometer (IMPLEN, CA, United States). DNA concentration was measured using Qubit^®^ DNA Assay Kit in Qubit^®^ 2.0 Fluorometer (LifeTechnologies, CA, United States). DNA sample was fragmented by sonication to a size of 350 bp, which were end polished, A-tailed, and ligated with the full-length adapter for Illumina sequencing with further PCR amplification. PCR products were purified (AMPure XP system) and libraries were analyzed for size distribution. These libraries were sequenced by using Illumina HiSeq platform and 150 bp paired-end reads were generated.

### BSA Analysis

Burrows-Wheeler Aligner (Heng and Richard, 2009) was used to align the clean reads of each sample against the reference genome. Alignment files were converted to BAM files using SAMtools software (Li et al., 2009). Variant calling was performed for all samples using the Unified Genotyper function in GATK3.3 (McKenna and Hanna, 2010) software. SNP was selected by using the Variant Filtration parameter in GATK and InDel was filtered by using the Variant Filtration parameter.

The reads depth information for the SNPs/InDels above in the offspring pools was gained to calculate the SNP/InDel index (Takagi and Abe, 2013). We filtered out those points whose SNP/InDel index in both pools were less than 0.3 and depth less than 7. The difference of the SNP/InDel index of two pools was calculated as the △ SNP/InDel index. The markers with an absolute value of △index between 0.69 and 1 were screened out as candidate molecular markers.

### Validation of Molecular Markers Related to Salt-Alkali Tolerance

SNPs and Indels conducted two rounds of verification (Table 1). The first round used a mixed template made by mixing the extracted DNA individual templates for PCR amplification and preliminary screening. The second round used individual DNA templates for amplification and checked by sequencing, or 2% agarose electrophoresis. Statistics of the genotype of each individual were based on the results of sequencing and electrophoresis. SPSS 17.0 software was used for one-way analysis of variance (ANOVA), and *p* < 0.05 was considered to be significantly related to salt-alkali tolerance.

**TABLE 1:** Primers used in this study.

Primer ID	Primer sequence (5′-3′)
S1-F	AGG​TCT​CCC​TCA​CAA​ACG​C
S1-R	TGT​GGA​TAG​GAA​AGG​GTG​AA
S2-F	CCA​TTA​CTT​ACT​CTC​ACC​ATT​TCA​G
S2-R	CAA​CCT​TGA​CGG​AAA​CCA​TAC
S3-F	CTC​ACT​TAC​CTG​TCG​TCA​CCT​G
S3-R	GCA​CGC​AGG​TAC​TGA​ACA​TTT
S4-F	ACC​ACA​CCT​GCC​TAA​TCT​ACC
S4-R	CAA​GAT​TCC​AGT​GTT​TCT​GTG​AG
S5-F	AGT​CAC​TAT​GAA​TGG​CAA​ATA​TCT​A
S5-R	CGG​TTT​GTA​ACT​CTC​GGG​GT
I4-F	CTC​TCC​GCC​AGC​CCG​CCA​TTA​ATG​C
I4-R	TTA​CTT​TCC​ATC​CAT​CAG​CC
I8-F	TGG​GTG​TTG​TCA​ATC​TGT​GAT​GGC​T
I8-R	CTG​ACT​GAC​GAG​ACG​ACT​GG
I9-F	TCG​TCA​GTC​ATC​TTT​CTT​CTC​TC
I9-R	TGT​TTG​GTT​ATT​GGC​GTT​G

### qPCR

The volume of the qPCR reaction system was 10 μL, containing 5 μL of 2 × SYBR Master Mix (RR420A, Takara Bio, Japan), 0.2 μL of each primer and ROX Reference Dye II, 1 μL of cDNA template, and 3.4 μL of rnase Free dH2O. qPCR was carried out in a FAST-7500 system (ABI-7500, ThermoFisher, Singapore) as follows: 95°C or 30 s, and 40 cycles of 95°C for 5 s and 60°C for 34 s. Results from qPCR were analyzed by SPSS software’s ANOVA for group differences, and Origin 9.1 was used for mapping.

## Results

### Screening of Candidate Molecular Markers

In this study, raw data of 45,854,625,300 and 49,159,475,100 were obtained from the populations with significant differences to salt-alkali tolerance (Table 2). The sequencing depth is 44.65X and 48.31X. A total of 855 SNPs (Supplementary Table S1) and 1051 Indels candidate polymorphic sex sites were selected with 95% confidence level (Supplementary Table S2). Among them, 395 were in the intron region and 1,420 in the intergenic region (Table 3).

**TABLE 2:** Summary of sequencing data quality.

Group	Raw data (bp)	Valid data after filtering (bp)	Efficient (%)	Error rate (%)	Q20 (%)	Q30 (%)	GC content%)
S	45,854,625,300	45,753,784,500	99.78	0.04	93.81	87.06	40.62
T	49,159,475,100	49,083,786,900	99.85	0.04	93.92	87.31	40.86

**TABLE 3:** Position information of candidate markers in the genome.

Category	Number of SNPs	Number of Indels
Upstream	12	27
Exonic-Stop gain	0	0
Exonic-Stop loss	0	0
Exonic-Synonymous	7	0
Exonic-Non-synonymous	2	0
Intronic	164	231
Splicing	0	0
Downstream	10	20
Upstream/Downstream	1	1
Intergenic	649	771
Ts	558	
Tv	297	
Ts/tv	1879	
Total	855	1051

### Validation of Molecular Markers

A total of ten SNPs and ten Indels were screened out based on △index value (close to 0 or 1). Using the mixed template for PCR amplification, a total of 20 sites were successfully amplified by the target PCR product. Then, 40 individuals were typed for the successfully verified markers in the mixed template by PCR product sequencing and gel agarose electrophoresis. The association analysis test was performed by SPSS, and eight of the markers showed significant correlation with salt-alkali tolerance (*p* < 0.05) (Table 4), including five SNPs and three Indels (Table 5).

**TABLE 4:** Genotyping information statistics.

Marker ID	Marker type	Genotype	Proportion of susceptible group	Proportion of tolerant group	*p*
S1	A/G	GG:AG:AA	18:2:0	4:10:6	0.000
S2	A/G	GG:AG:AA	12:6:2	20:0:0	0.007
S3	A/G	GG:AG:AA	15:1:0	6:10:0	0.001
S4	T/C	CC:CT:TT	11:7:2	2:11:7	0.007
S5	A/G	GG:AG:AA	0:3:17	12:0:8	0.000
I4	Indel	In:Del:Indel	0:15:5	13:5:2	0.000
I8	Indel	In:Del:Indel	5:2:13	18:0:2	0.000
I9	Indel	In:Del:Indel	7:4:9	0:16:4	0.000

**TABLE 5:** Annotation information of salt-alkali tolerance candidate genes.

Gene ID	Category	△index	Annotation
Contig 574.9	Intergenic	−0.92	NR:Ubiquitin-conjugating enzyme E2 O [Zootermopsis nevadensis]
			KEGG:ubiquitin-conjugating enzyme E2-230k, putative (EC:6.3.2.19)
			K10581 ubiquitin-conjugating enzyme E2 O [EC:2.3.2.24] (A)
			SwissProt:E2/E3 hybrid ubiquitin-protein ligase UBE2O OS = Mus musculus GN = Ube2o
			PE = 1 SV = 3; IPR_id:NULL; IPR_Anno:NULL
Contig 11.37	Intergenic	0.74	NR:hypothetical protein X975_00935, partial [Stegodyphus mimosarum]
			KEGG:putative ZDHHC-type palmitoyltransferase 6; K20032 palmitoyltransferase ZDHHC13/17 [EC:2.3.1.225] (A); SwissProt:Espin OS = Mus musculus GN = Espn PE = 1 SV = 2
			IPR_id:NULL; IPR_Anno:NULL
Contig 272.2	Intergenic	0.71	NR:PREDICTED: aspartate--tRNA ligase, mitochondrial [Tribolium castaneum]
			KEGG:kinesin-6A; kinesin 6A; K17387 kinesin family member 23 (A)
			SwissProt:Aspartate--tRNA ligase, mitochondrial OS = Rattus norvegicus GN = Dars2 PE = 1 SV = 1
			IPR_id:IPR008126; IPR_Anno:Outer membrane adhesion, Yersinia
Contig 159.21	Intergenic	0.78	NR:PREDICTED: gamma-tubulin complex component 6 isoform X2 [Pogonomyrmex barbatus]
			KEGG:TUBGCP6; tubulin, gamma complex associated protein 6
			K16573 gamma-tubulin complex component 6 (A)
			SwissProt:Gamma-tubulin complex component 6 OS = Homo sapiens
			GN = TUBGCP6 PE = 1 SV = 3; IPR_id:IPR007259; IPR_Anno:Spc97/Spc98
Contig 105.16	Intergenic	0.79	NR:PREDICTED: uncharacterized protein LOC105387196 [Plutella xylostella]
			KEGG: ; SwissProt: ; IPR_id:NULL; IPR_Anno:NULL
Contig 48.16	Intergenic	−0.77	NR: ; KEGG: ; SwissProt:Transcription termination factor 1 OS = Homo sapiens
			GN = TTF1 PE = 1 SV = 3; IPR_id:IPR005448; IPR_Anno:Voltage-dependent calcium channel, P/Q-type, alpha-1 subunit
Contig 2378.3	Intergenic	0.75	NR:PREDICTED: vesicle-trafficking protein SEC22b-B [Fopius arisanus]
			KEGG:vesicle-trafficking protein SEC22b-B; K08517 vesicle transport protein SEC22 (A)
			SwissProt:Vesicle-trafficking protein SEC22b-B OS = Danio rerio
			GN = sec22bb PE = 2 SV = 1; IPR_id:IPR002255; IPR_Anno:Flavin monooxygenase (FMO) 3
Contig 370.5	Intergenic	−0.72	NR:PREDICTED: protein SMG5 [Acromyrmex echinatior]
			KEGG:protein SMG5; K11125 protein SMG5 (A)
			SwissProt:Protein SMG5 OS = Homo sapiens GN = SMG5 PE = 1 SV = 3
			IPR_id:IPR018834; IPR_Anno:DNA/RNA-binding domain, Est1-type

### Enrichment Analysis of Candidate Genes

**FIGURE 1:**
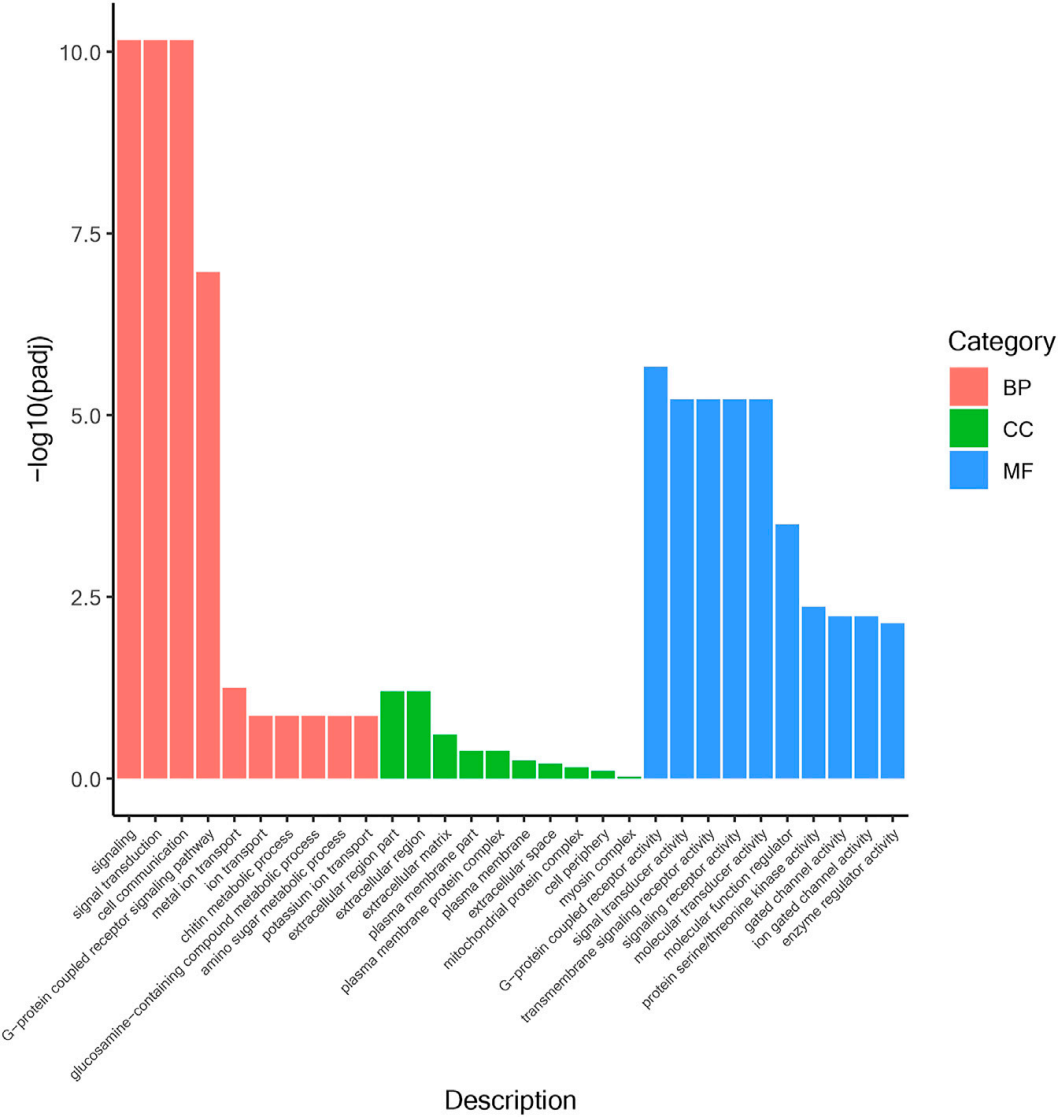
GO enrichment analysis of salt-alkali tolerance candidate genes.

**FIGURE 2:**
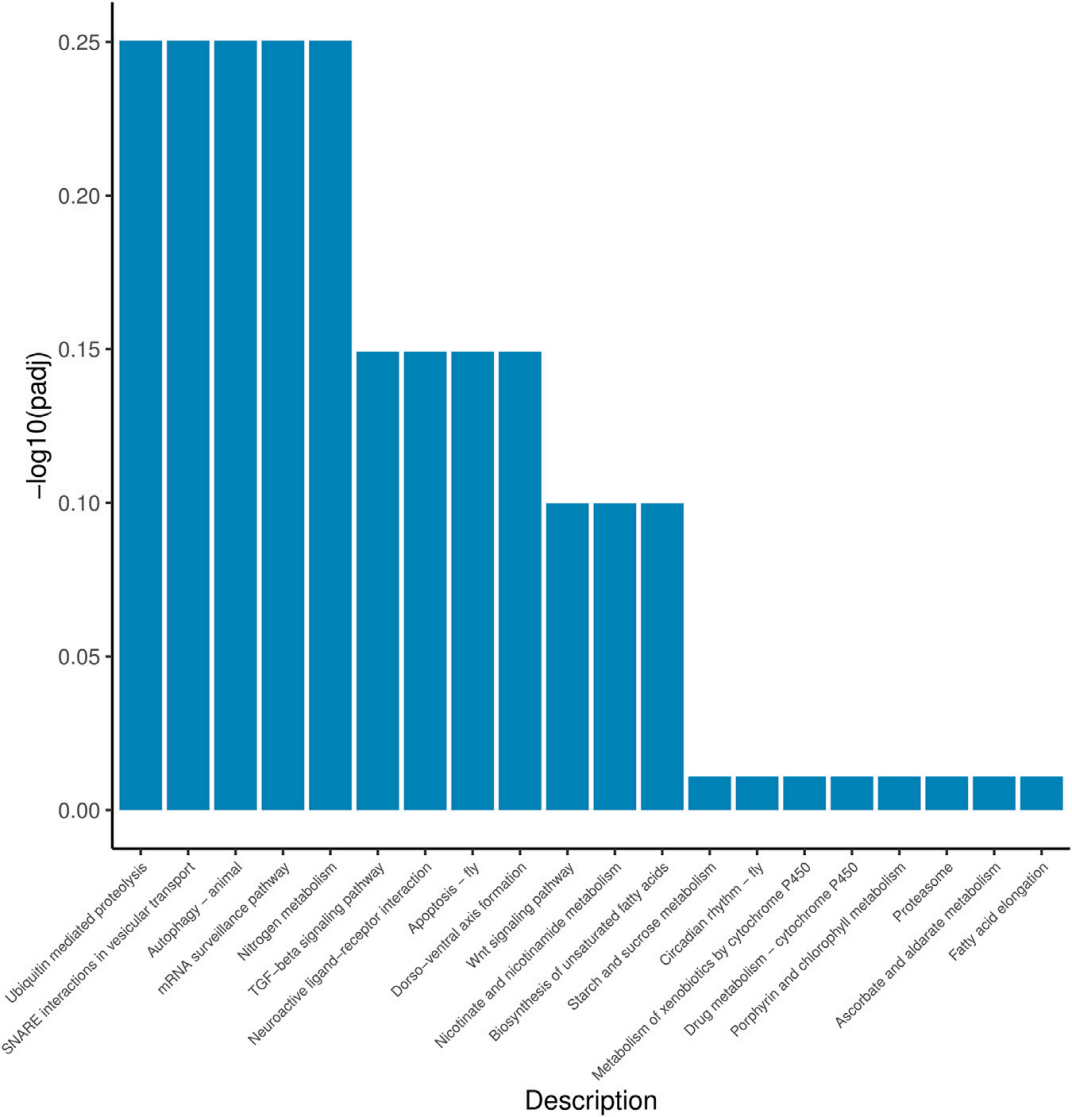
KEGG enrichment analysis of salt-alkali tolerance candidate genes.

According to the position information of the candidate marker in the genome, a total of 2171 genes were located, which were used to perform Gene Ontology (GO) and Kyoto Encyclopedia of Genes and Genomes (KEGG) enrichment analyses. The GO results (Figure 1) showed that 30 processes were significantly enriched, of which “signaling”, “extracellular region part”, and “G-protein coupled receptor activity” were the main processes with the largest number of genes in “Biological Process”, “Cellular Component” and “Molecular Function”, respectively. Besides, a total of 20 KEGG pathways (Figure 2) were enriched, among which “Ubiquitin mediated proteolysis”, “SNARE interactions in vesicular transport”, “Autophagy”, “mRNA surveillance pathway” and “Nitrogen metabolism” were the most enriched pathways (*p* < 0.05).

### Expression Analysis of Candidate Genes

**FIGURE 3:**
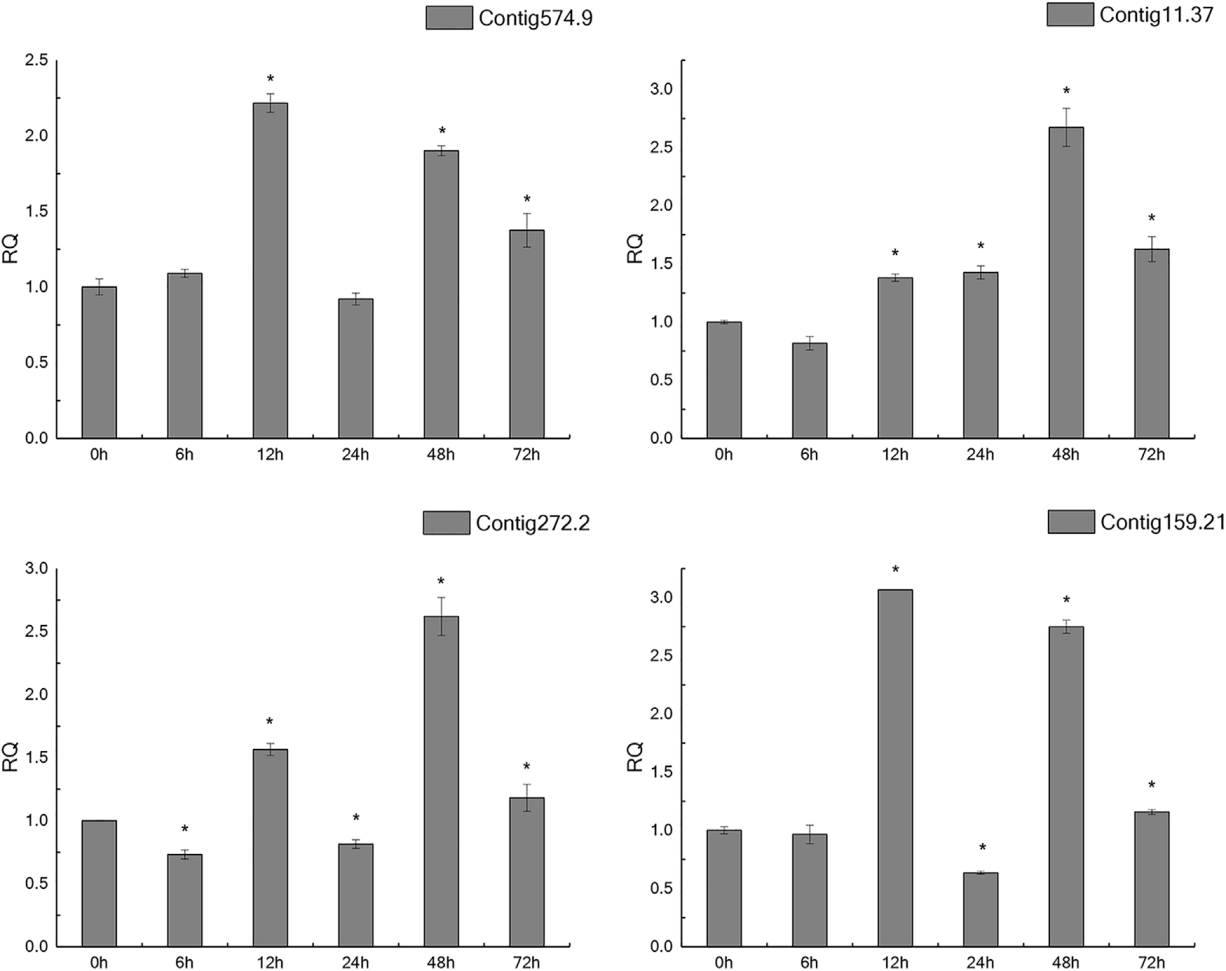
qPCR results of salt-tolerant-related genes.

**FIGURE 4:**
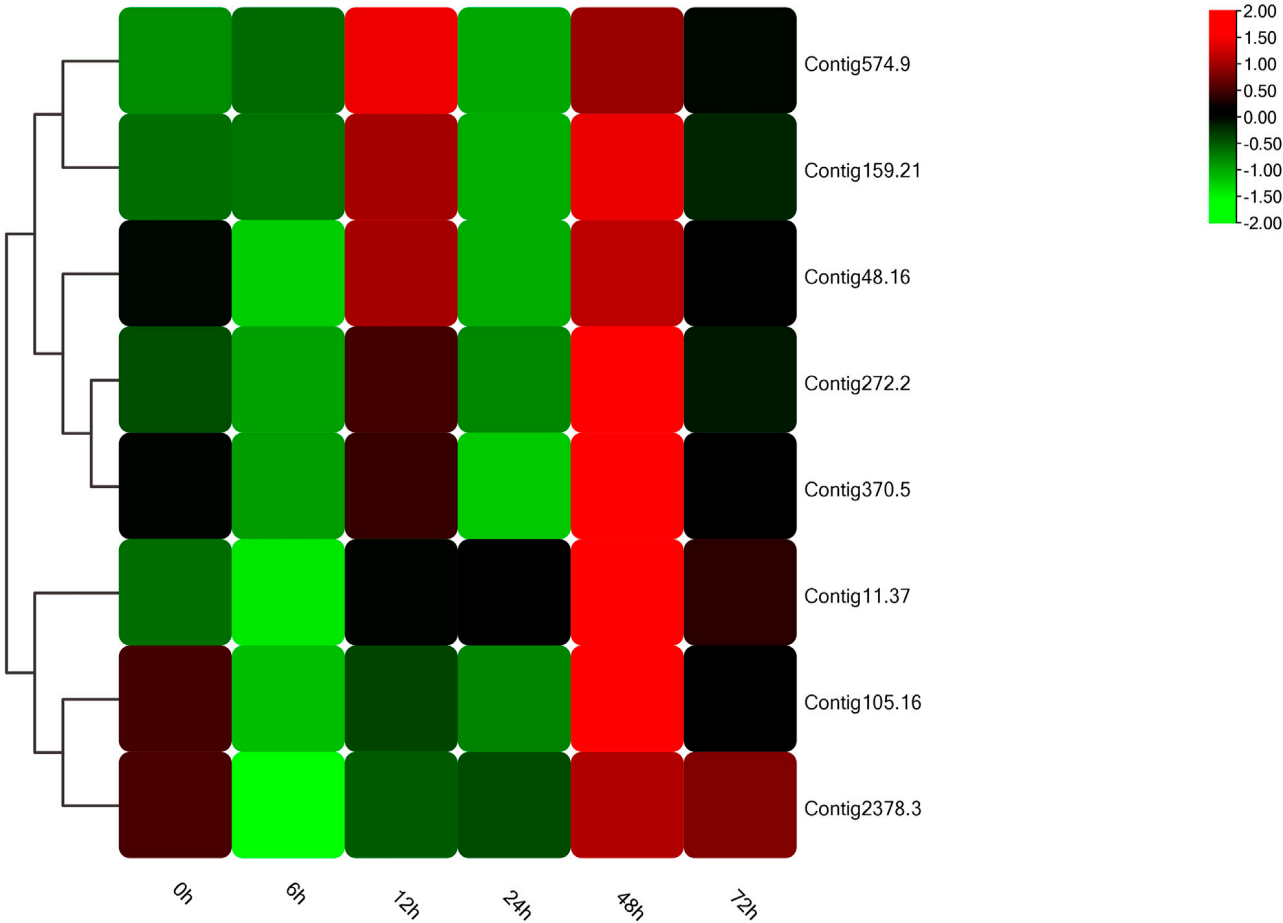
Heat map of salt-tolerant-related genes.

In the light of gene annotation and enrichment analysis results, eight genes related to proteolysis, autophagy, nitrogen metabolism, and vesicle transport were screened. qPCR results showed that the expression patterns of these genes changed significantly after salt-alkali stress (Figure 3), suggesting that they play a role in the adaptation to salt-alkali. The results show that these genes can be divided into two types (Figure 4). One group was significantly up-regulated at 48 h and the other group of genes was significantly up-regulated at 12 and 48 h with a trend of fluctuating expression.

## Discussions

In this study, we successfully screened the salt-alkali tolerance markers in *P. trituberculatus,* using the BSA strategy for the first time, and then anchored several genes related to salt-alkali tolerance. The BSA method that was proposed by Michelmore and Paran, (1991) can lock the target trait genes or markers by comparing the degree of difference in the allele frequency at the polymorphic site between the two groups with markedly differentiated traits (June 2021; Rujia and Yiming, 2021; Zaiqing and Anmin, 2021). At present, research on trait-related markers of aquatic animals is mainly based on QTL and association analysis (Wang and Liu, 2017; Cao and Wang, 2021). QTL analysis relies on pedigree materials, and only excavated markers are applicable (Zhixue and Khorshed, 2021). Association analysis is often based on re-sequencing and the cost is relatively high (Hussain and Rivandi, 2007; Vermerris, 2011; Ali, 2013). Compared with the above two methods, BSA has the advantages of being able to use population materials and being cost effective and time efficient (Takagi Abe, 2013). Using the BSA strategy, trait-related markers have been successfully developed in plants (Cheng and Abass, 2020; Marc, 2020; Farhan and Qiutao, 2021; Hossein and Azin, 2021), proving it to be a feasible and economical method. However, its applicability in aquatic animals had not been reported in the past. In this study, 20 markers were selected based on BSA analysis, and eight salt-alkali tolerance related markers were finally developed efficiently. The results of the study show that BSA strategy is suitable for the mining of trait-related markers in aquatic crustaceans.

We noticed that most of the successfully verified SNPs and Indels are located on the intergenic region (81.82%). The intergenic region was once considered unimportant. However, in recent years, it has been found that these regions contain functionally important elements (Cai, 2016) and independent transcription of miRNAs with their own promoters (Hess et al., 2006). Intergenic regions may also contain unidentified functional elements, such as non-coding RNA (Zhong et al., 2014; Fariha and Changrui, 2019). It is currently believed that intergenic regions can control gene expression and inhibition, and help RNA to identify genes with low conservation and diversity (Ingvarsson et al., 2016; Bakhtiarizadeh and Salehi, 2018). The fact that most of the saline-alkali markers are located in the intergenic region, indicates that some potential functional regions of the intergenic region may have important functions in the regulation of saline-alkali adaptation. However, the specific mechanisms are still unclear and necessitate further research.

Based on the location information of markers in the genome, we initially anchored 2171 potential saline-alkali adaptation genes, which were mainly enriched in the pathway including ubiquitin-mediated proteolysis, SNARE interactions in vesicle transport and autophagy. These results indicate that the mechanism of saline-alkali adaptation is complex and co-regulated by an equally complex regulatory network. It is worth noting that the salt-alkali-related ion transport genes such as Na^+^-K^+^-ATPase are not included here. This suggests that, although ion transport plays an important role in the process of saline-alkali adaptation, it does not play a prominent role in the differentiation of salt-alkali tolerance.

In addition, according to annotation information, 8 genes were screened from the five most enriched KEGG pathways for qPCR verification. The expression patterns of these eight genes changed significantly after salt-alkali stress, indicating that they may have certain functions in salt-alkali adaptation, or salt-alkali stress has affected their normal functions. We also found that most genes were up-regulated at 12 and 48 h, suggesting the critical time for salt-alkali adaptation.

In this study, we screened the salt-tolerant markers to prove the feasibility of the BSA strategy in aquatic research. The 8 successfully verified markers provide usable markers for subsequent molecular marker-assisted breeding. Furthermore, this experiment anchored some candidate genes related to salt-alkali tolerance. These results will help to systematically clarify the molecular mechanism of salt-alkali adaptation of *P. trituberculatus*.

## Data Availability

The original contributions presented in the study are publicly available in NCBI using accession number PRJNA755748.
